# Synergistic Effects of Active Sites’ Nature and Hydrophilicity on the Oxygen Reduction Reaction Activity of Pt-Free Catalysts

**DOI:** 10.3390/nano8090643

**Published:** 2018-08-22

**Authors:** Mariangela Longhi, Camilla Cova, Eleonora Pargoletti, Mauro Coduri, Saveria Santangelo, Salvatore Patanè, Nicoletta Ditaranto, Nicola Cioffi, Anna Facibeni, Marco Scavini

**Affiliations:** 1 Dipartimento di Chimica, Università degli Studi di Milano, Via Golgi 19, 20133 Milano, Italy; camilla.cova@studenti.unimi.it (C.C.); eleonora.pargoletti@unimi.it (E.P.); marco.scavini@unimi.it (M.S.); 2 ESRF—The European Synchrotron, 71, Avenue des Martyrs, 38043 Grenoble, France; mauro.coduri@esrf.fr; 3 Dipartimento di Ingegneria Civile, dell’Energia, dell’Ambiente e dei Materiali, Università “Mediterranea”, Via Graziella, Loc. Feo di Vito, 89122 Reggio Calabria, Italy; saveria.santangelo@unirc.it; 4 Dipartimento di Scienze Matematiche e Informatiche, Scienze Fisiche e Scienze della Terra, Università di Messina, Viale Stagno d’Alcontres 31, 98166 Messina, Italy; salvatore.patane@unime.it; 5 Dipartimento di Chimica, Università degli Studi di Bari Aldo Moro, Via Orabona 4, 70125 Bari, Italy; nicola.cioffi@uniba.it (N.D.); nicoletta.ditaranto@uniba.it (N.C.); 6 Dipartimento di Energia and NEMAS—Centre for NanoEngineered MAterials and Surfaces, Politecnico di Milano, Via Ponzio 34/3, 20133 Milano, Italy; anna.facibeni@polimi.it

**Keywords:** oxygen reduction reaction, Pt-free catalysts, CNT N-doped carbons, active site hydrophilicity

## Abstract

This work highlights the importance of the hydrophilicity of a catalyst’s active sites on an oxygen reduction reaction (ORR) through an electrochemical and physico-chemical study on catalysts based on nitrogen-modified carbon doped with different metals (Fe, Cu, and a mixture of them). BET, X-ray Powder Diffraction (XRPD), micro-Raman, X-ray Photoelectron Spectroscopy (XPS), Scanning Electron Microscopy (SEM), Scanning Transmission Electron Microscopy (STEM), and hydrophilicity measurements were performed. All synthesized catalysts are characterized not only by a porous structure, with the porosity distribution centered in the mesoporosity range, but also by the presence of carbon nanostructures. In iron-doped materials, these nanostructures are bamboo-like structures typical of nitrogen carbon nanotubes, which are better organized, in a larger amount, and longer than those in the copper-doped material. Electrochemical ORR results highlight that the presence of iron and nitrogen carbon nanotubes is beneficial to the electroactivity of these materials, but also that the hydrophilicity of the active site is an important parameter affecting electrocatalytic properties. The most active material contains a mixture of Fe and Cu.

## 1. Introduction

An oxygen reduction reaction (ORR) is a fundamental step in many electrochemical applications, e.g., fuel cells and zinc/air batteries. Being kinetically hindered, an ORR requires efficient catalysts typically based on Platinum Group Metals [1,2,3]. These materials, though very efficient, present some drawbacks, such as costs, availability, and technological problems. Finding cheaper substitutes with similar or better electrocatalytic properties and stability is a challenge for the scientific community [4,5,6,7,8,9,10,11,12,13,14,15,16,17]. Effective candidates are based on metal–nitrogen–carbon catalysts, in which the metal is usually iron or cobalt [4,5,6,7,8,9,10,11]. Many studies have been devoted to highlighting the complex nature and chemical environment of catalytic sites [12,13,18]. Principal results show that several types of active sites are present on the catalyst surface [14], and that iron coordination complexes with nitrogen promote ORRs to water [15]. Moreover, all these active centers are embedded in graphene planes [19]. Recently, by using N-doped carbon catalysts with supported and not embedded metal centers with different natures, specific surface areas, types, and amounts of surface nitrogen, it has been proposed that the hydrophilicity of the catalyst support has an important role on ORR activity [20]. In fact, a high hydrophilicity allows for a high dispersion of metal-containing active sites and a better accessibility of oxygen to catalytic centers [20]. However, a recent work has shown that, for nanoporous nitrogen-doped carbons, the adsorption of water molecules depends on the local density of nitrogen atoms rather than their nature [21]. Therefore, the simultaneous use of different hydrophilic supports, nitrogen amounts, and specific surface areas [20] could lead to cumulative effects, masking the importance of hydrophilicity either of the supporting carbon, as stated in [20], or of the active centers.

The aim of this work is to demonstrate the importance of the hydrophilicity of the active sites for ORRs. For this purpose, an electrochemical and physico-chemical study was carried out on catalysts based on nitrogen-modified carbon doped with different metals (Fe, Cu, and a mixture of them), having a comparable specific surface area and amount of nitrogen to avoid hiding cumulative effects.

The studied catalysts were synthesized by thermal decomposition of a mixture of a sugar, guanidine acetate, metal salts, and silica as templating agent. In the following, catalysts containing Fe, Cu, and Fe–Cu will be respectively labelled S_GA_Fe, S_GA_Cu, and S_GA_FeCu, whereas S_GA will indicate a metal-free (reference) sample.

## 2. Materials and Methods

### 2.1. Catalyst Preparation

All chemicals/solvents were purchased from Sigma Aldrich (Milano, Italy) and used as received without further purification. Gelling sugar (Südzucher AG, Mannheim, Germany) was purchased in a market (sucrose:pectin = 98%:2%, from NMR). A Pt-based commercial catalyst (EC20, 20% Pt/carbon) was tested and used as a reference material.

In the synthesis, gelling sugar (3 g), guanidine acetate (2 g), and metal (Me) acetate (1 wt.% total metal ion calculated on the total mass of sugar and guanidine acetate, Me = Fe, Cu, or a Fe:Cu = 0.5 wt.%:0.5 wt.% mixture) were added to silica powder (4.3 g) and mixed. This mixture underwent a first heating step (T = 600 °C, 1 h, N_2_ flow rate = 100 cm^3^ min^−1^) followed by lixiviation in boiling NaOH (3 mol dm^−3^) to remove silica. After washing, the carbon was dried (T = 110 °C, 24 h, N_2_) and, finally, pyrolysed (T = 900 °C, 3 h, N_2_ flow rate = 100 cm^3^ min^−1^) to activate catalytic sites.

### 2.2. Physical Characterizations

Specific surface area and porosity distribution were obtained from N_2_ adsorption/desorption isotherms at 77 K using a Micromeritics Tristar II 3020 (Micromeritics, Milano, Italy) apparatus and the instrumental software (Version 1.03) and applying Brunauer–Emmett–Teller (BET) and Barrett–Joyner–Halenda analyses, respectively. Prior to measuring, sample powders were heat-treated (T = 150 °C, 4 h, N_2_) to remove adsorbed foreign species.

Micro-Raman spectra were recorded in air at room temperature using a NTEGRA-Spectra SPM spectrometer (NT-MDT, Moscow, Russia) (350 mm monochromator, MS3504i, ANDOR Idus CCD cooled at T = −60 °C). Raman scattering was excited by a thermo-cooled solid-state laser (λ = 532 nm; 2.33 eV). The scattered light from the sample was collected using a 100x (0.75 Numerical Aperture) Mitutoyo objective. Spectra acquired from different random positions on each specimen were averaged to have a reliable picture of the sample bulk. The average spectra were analyzed by using a commercially available software package [22] to assess the graphitization degree of samples.

X-ray Photoelectron Spectroscopy (XPS) analyses were run on a PHI 5000 Versa Probe II Scanning XPS Microprobe spectrometer (ULVAC-PHI Inc., Kanagawa, Japan). Measurements were done with a monochromatic Al Kα source (X-ray spot 100 μm) at a power of 24.8 W. Wide scans and detailed spectra were acquired in Fixed Analyzer Transmission (FAT) mode with a pass energy of 117.40 eV and 29.35 eV, respectively. An electron gun was used for charge compensation (1.0 V 20.0 μA). Data processing was performed by using the MultiPak software v. 9.5.0.8 (ULVAC-PHI Inc., Kanagawa, Japan).

X-ray Powder Diffraction (XRPD) patterns were collected at room temperature at the ID22 beamline of the ESRF with a wavelength λ = 0.399946(4) Å up to 2θ = 40° and a ~0.5 h/pattern counting time. Data recorded continuously have been then merged using different step sizes spanning from Δ2θ = 0.005° to 0.050° to highlight different features in the patterns. Patterns merged with the former step size were analyzed via the Rietveld method using the General Structure Analysis System (GSAS) suite of the program [23].

Scanning Electron Microscopy (SEM) observations were carried out using a Leo 1430 SEM (Zeiss, Oberkochen, Germany).

Scanning Transmission Electron Microscopy (STEM) measurements were performed by using a Zeiss Supra 40 Field Emission Scanning Electron Microscope (FE-SEM) (Zeiss, Oberkochen, Germany) working in high vacuum conditions and equipped with the GEMINI column.

Wettability features of carbons were evaluated by a Krüss Easy Drop instrument (Krüss, Hamburg, Germany) using a water drop (V = 7 μL) gently placed on the surface of the samples compacted as a flat layer. By taking into account the complexity of the system and the relationship between the wetting properties and the physico-chemical features of the materials (the size of particles, the roughness of the samples, and their intrinsic surface free energy and packing degree) only the time necessary to achieve complete spreading was measured [20].

Electrochemical characterization was performed in 0.1 mol dm^−3^ KOH by the Thin Film Rotating Disk Electrode method using Cyclic Voltammetry (CV) as in [16]. ORR onset potential was evaluated at j = 1 mA cm^−2^ and the number of exchanged electrons was determined as described in Ref. [16].

The stability of materials was evaluated by comparing CV curves at 1600 rpm in O_2_ at the beginning and at the end of electrochemical measurements (results not reported). In all cases, the stability was good.

## 3. Results and Discussion

**Figure 1:**
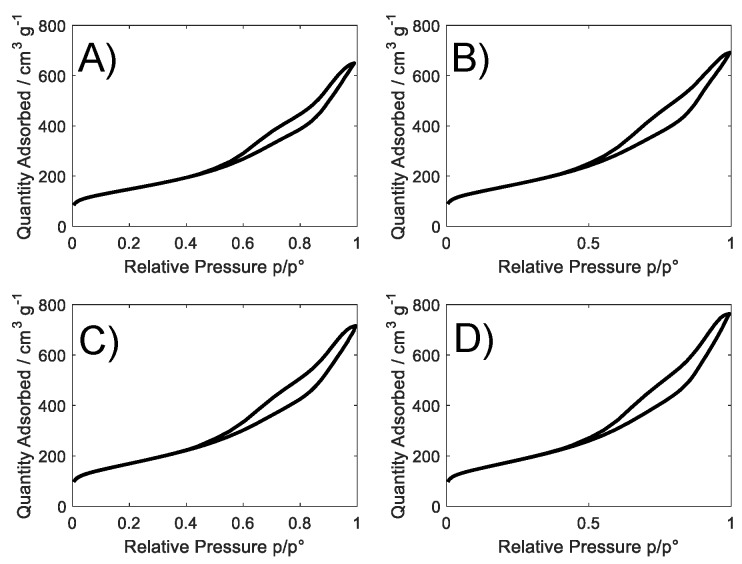
Adsorption and desorption nitrogen isotherms for (**A**) S_GA; (**B**) S_GA_Cu; (**C**) S_GA_Fe; and (**D**) S_GA_FeCu.

Figure 1 shows experimental BET adsorption isotherms. All the samples exhibit a type IV isotherm with a H3-type hysteresis loop [24] characteristic of mesoporous materials and attributable to a non-fixed aggregation of plate-like particles giving rise to slit-shaped pores [25].

The specific surface area (Table 1) of samples is between ~520 and 600 m^2^ g^−1^ without important differences among metal-doped samples (~560–600 m^2^ g^−1^, with a difference of 7%). A high mesoporosity (~55%), centered between 2 and 5 nm, characterizes all these materials (Table 1 and Figure S1). ORRs benefit from this pore range, since it favors the diffusion of reactants/products to/from active centers [26,27,28].

**Figure 2:**
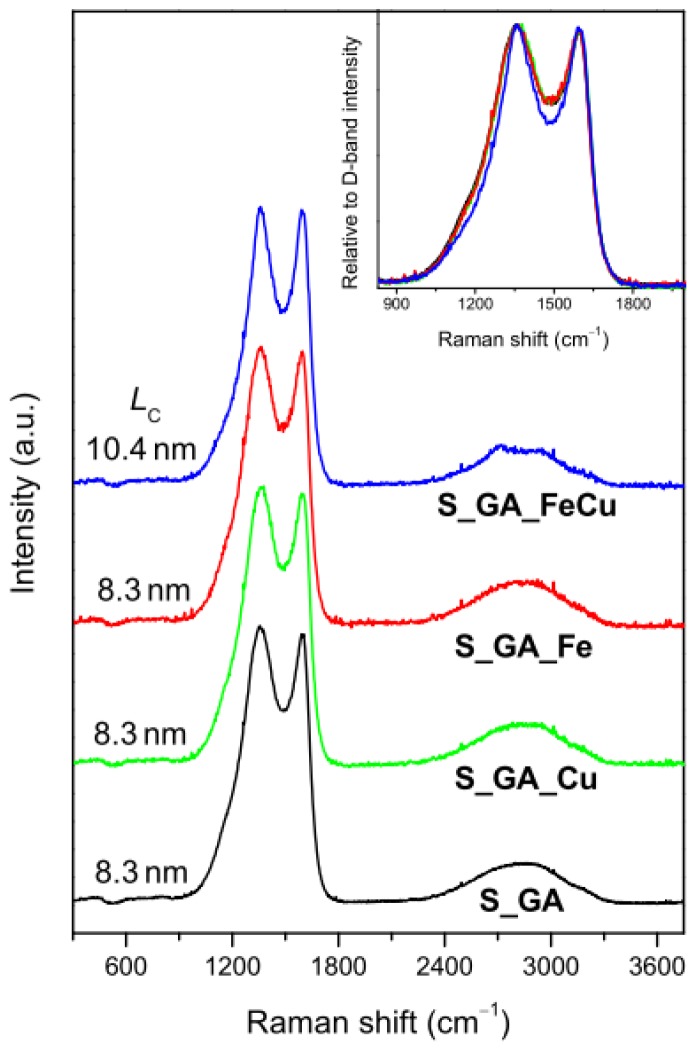
Micro-Raman spectra of the investigated catalysts (the average size of the graphitic crystallites (*L*_C_), estimated from the G/D intensity ratio [31] is reported). Inset: comparison of the D- and G-band regions of the spectra.

The influence of a metal precursor on the crystalline arrangement of the carbon phase in the catalysts was investigated by means of micro-Raman spectroscopy. Regardless of the presence and type of the metal, these materials exhibit the Raman profile peculiar to highly disordered graphitic carbons (Figure 2). Two very intense bands peaking at ~1360 cm^−1^ and ~1590 cm^−1^ dominate the lower frequency region (<2000 cm^−1^) of the spectra. The band peaking at ~1360 cm^−1^ (D-band) is associated with the in-plane breathing mode of the C hexagonal rings [29]; the band peaking at ~1590 cm^−1^ (G-band) originates from the stretching of C=C pairs [29]. Moreover, a weaker and broader featureless second-order structure, typical of highly disordered carbon nanostructures, is detected at ~2700 cm^−1^.

Since the D-band is generated by finite size effects and by lattice defects breaking the translational symmetry of graphitic layers [29], while the G-band is the fingerprint of the graphitic crystalline arrangement, the G/D integrated intensity ratio (IG/ID) is commonly regarded as a graphitization index [30] (the higher its value, the higher the graphitization degree of the sample).

No evident difference is observed in the lower frequency region of the spectra (inset of Figure 2) [22,29,30], but a slight D-band shrinking in the spectrum of S_GA_FeCu was observed. This hints at a little influence of both the presence and type of the metal on the crystalline arrangement of the carbon phase. The results of spectra decomposition, reported in detail in the Supplementary Material (Figure S2, Table S1), show that the highest graphitization degree (i.e., the greatest IG/ID value, 0.55 against 0.43–0.44) pertains to S_GA_FeCu. In this sample, the D-band is narrower than in the other metal-containing catalysts (183 against 211−215 cm^−1^) and the average size of the graphitic crystallites, estimated from the relationship proposed by Cançado et al. [31] for nanographites, is larger (10.4 against 8.3 nm).

**Figure 3:**
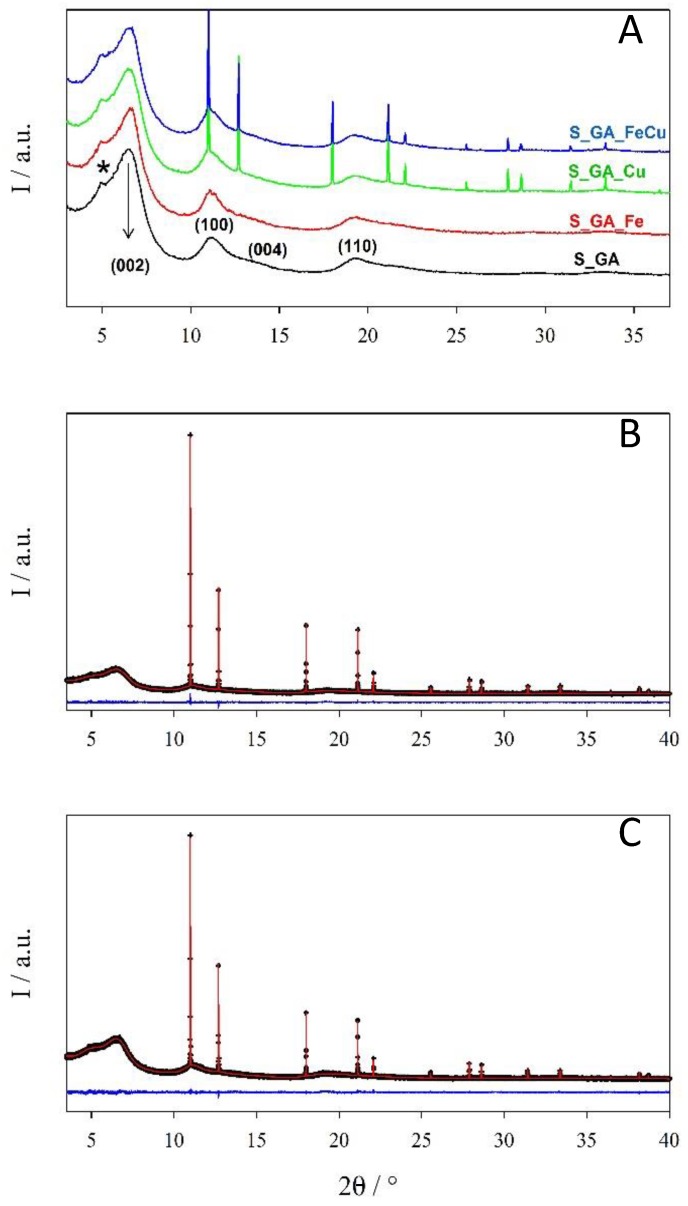
(**A**) XRPD patterns merged using a Δ2θ = 0.050° step size highlighting the graphitic contribution. Numbers in brackets are the Miller indexes of the hexagonal graphite phase. A star highlights a broad bump due to the kapton capillary. Rietveld refinements for samples (**B**) S_GA_Cu and (**C**) S_GA_FeCu. Observed (crosses) and calculated (continuous line) profiles and residuals (bottom).

The results of XRPD analysis (Figure 3) fully agree with this picture. Despite the presence of different metals, all samples show broad signals at ~6.5, 11.1°, 13.3°, and 19.3° related to disordered graphitic carbon [16]. To highlight these peaks, in this figure patterns with a Δ2θ = 0.050° step size are shown with the corresponding Miller (hkl) indexes (Figure 3A). These signals are the only ones present in S_GA and S_GA_Fe patterns; conversely, in the other samples, Bragg peaks prove the presence of crystalline metal phases with a crystallite size of the order of ~400 nm. Rietveld refinements reveal that both S_GA_Cu and S_GA_FeCu patterns (Figure 3B,C) are suitably fitted by using one single ccp structure (space group Fm–3m) with sharp peaks and a carbon graphitic one (space group P63mc) where only a few broad peaks are apparent. In this case, a Δ2θ = 0.005° step size was adopted due to the sharpness of peaks of the metal phase.

The refined parameters are reported in Table S2. The lattice constants of the Fm–3m phase are 3.61711(1) and 3.61774(1) Å for S_GA_Cu and S_GA_FeCu, respectively. Though the difference is very small, the high resolution of the ID22 instrument makes it significant. Such a small expansion observed in S_GA_FeCu is consistent with a Cu-rich Cu1-xFex solid solution [32] as observed for xCu ≅ 0.03 at a T = 900 °C calcination temperature [33]. Biphasic Rietveld refinements show that the weight fraction of the metal phase is 2.6% and 1.6% in S_GA_Cu and S_GA_FeCu samples, respectively. We must warn the reader that, due to the noise in the broad peaks of the graphitic carbon peaks and to some arbitrariness in background subtraction, this phase analysis has to be considered as semi-quantitative. However, it points to an almost iron-free crystalline phase in the S_GA_FeCu sample. As in the case of S_GA_Fe one, most of the iron does not form extended crystalline phases.

**Figure 4:**
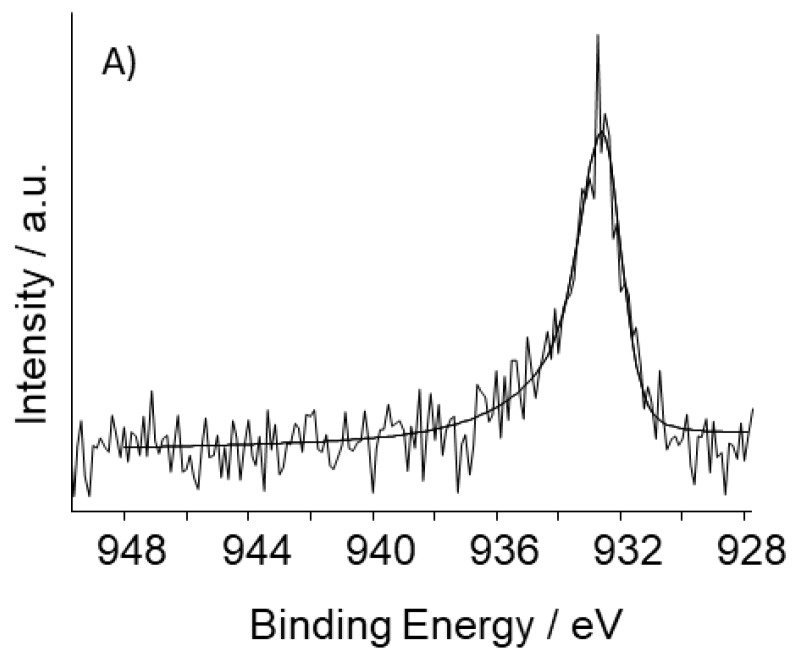

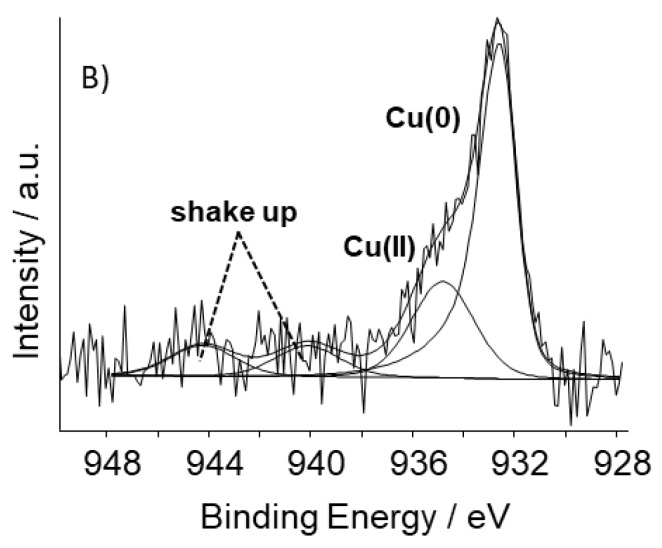
High-resolution photoelectron spectra of the Cu2p^3/2^ core level of: (**A**) S_GA_FeCu; (**B**) S_GA_Cu.

Figures S3–S6 and Figure 4A,B report XPS results. The concentration of metal species on the surface ranges between 0.2 and 0.4%. Nitrogen and oxygen species are detected in all the samples (Figure S3, Table 2), but their concentration depends neither on the presence nor on the nature of the metal (Table 2), though, according to data in the literature, it would be expected to vary with the specific metal used in the synthesis [34]. This singular behavior, combined with the picture emerging from the micro-Raman and XRPD analyses, evidences the peculiarity of the present materials with respect to those generally described in the literature.

The analysis of the high resolution photoelectron spectra of the N1s core level (Figure S4 and Table 3) evidences different types of nitrogen species on the surface of carbon [19,35]. Pyridinic, pyrrolic, and N_x_Me nitrogen are the most important for ORR electroactivity [19]. Considering standard deviation, no appreciable differences among metal-doped samples are observed.

In Figure S5, high resolution spectra of the C1s core level for all synthesized samples are reported. It can be observed that in the range of the explored binding energies, all the spectra are overlapped. Therefore, the total amount of carbon-oxygen functionalities (C_x_O_y_) on the carbon surface, considered as a measure of defects or edge sites in the graphene-like network [19], and the sp^2^/sp^3^ ratio related to the graphitization degree [36], are the same for all materials. These data further confirm the results reported above.

The high resolution photoelectron spectrum of the Cu2p^3/2^ core level in S_GA_FeCu (Figure 4A) can be fitted to a single peak attributable to Cu(0), while in S_GA_Cu, in addition to shake-up satellite peaks, two contributions assignable to Cu(0) and Cu(II) are found (Figure 4B) [37]. The absence of Cu(II) in S_GA_FeCu could be due to a Cu(II) surface concentration lower than the detection limit. On the other hand, the standard potentials of the redox couples Cu^2+^/Cu and Fe^2+^/Fe (E°Cu^2+^/Cu = +0.34 V/SHE and E°Fe^2+^/Fe = −0.44 V/SHE), suggest that the presence of iron during the synthesis lowers the stability of Cu(II) species.

The low signal-to-noise ratio of the Fe2p high resolution regions (Figure S6) might make unreliable the results of the curve-fitting procedure. Nevertheless, the main peak is centered at the same binding energy (B.E. = ~710 eV) in the two samples.

**Figure 5:**
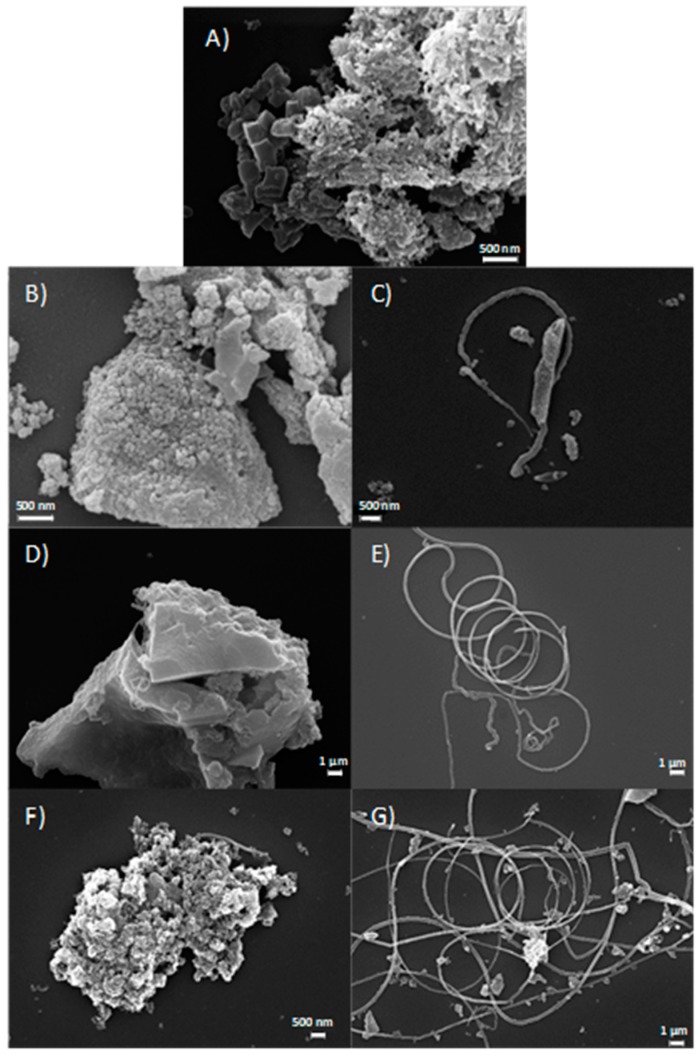
SEM images: (**A**) S_GA; (**B**,**C**): S_GA_Cu; (**D**,**E**): S_GA_Fe; (**F**,**G**): S_GA_FeCu.

Figure 5A–G report SEM images of the samples. In accordance with the porosity distribution results, all carbons are characterized by a porous structure (Figure 5A,B,D,F) due to the use of a hard templating agent lixiviated at the end of the synthetic process [38]. Interestingly, with respect to the other synthetic path [39], filamentous nanotubes are also evidenced in metal-doped samples (Figure 5C,E,G). In iron-doped carbons, these nanostructures are in a larger amount, are more well-organized, and longer than in S_GA_Cu. The high catalytic activity of iron to form ordered carbon nanostructures [40] might account for this behavior.

**Figure 6:**
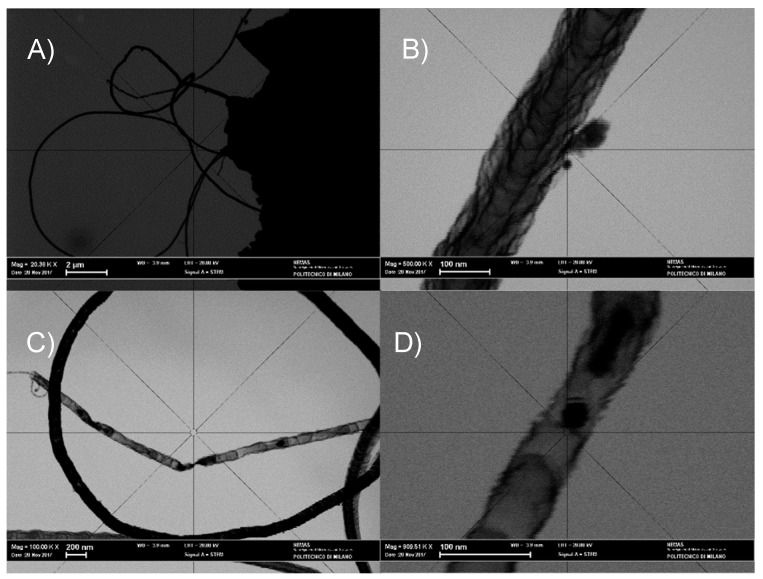
STEM images of S_GA_Fe. (**A**) Overview; (**B**–**D**) Details of (**A**).

The results of STEM analysis shown in Figure 6A–D corroborate the picture emerging from the SEM observations and reveal that the filaments present in the sample S_GA_Fe are featured by a bamboo-like structure peculiar to N-doped carbon nanotubes (N-CNTs). A few nanoparticles (~20–30 nm in diameter) are entrapped within the nanotubes. Since XRPD does not evidence any Bragg peaks related to iron metal phases (Figure 3B,C), if crystalline, their concentration should be below the detection limit of our synchrotron radiation diffraction measurements.

**Figure 7:**
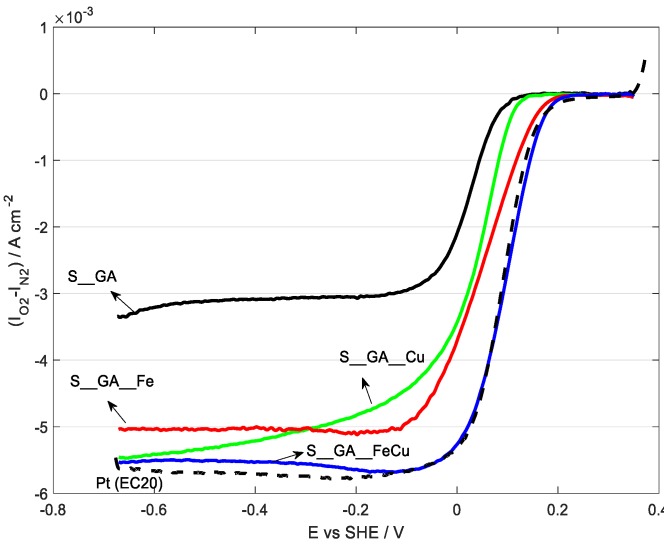
Oxygen reduction reaction (ORR) polarization curves recorded in oxygen-saturated 0.1 M KOH, v = 5 mV s^−1^, ω = 1600 rpm, T = 25 °C.

Polarization curves in 0.1 mol dm^−3^ KOH are reported in Figure 7 and elaborated data are displayed in Table 4. A polarization curve for a commercial catalyst based on Pt (EC20, 20% Pt) and relative data are also shown for comparison.

Except for S_GA_Cu, all the samples show a well-defined limiting current. The best electrocatalytic performance pertains to S_GA_FeCu, with the most anodic onset potential (0.145 V versus SHE) and the highest *E*_1/2_ (0.099 V versus SHE), also considering the Pt-based catalyst (0.135 V versus SHE and 0.088 V versus SHE, respectively). S_GA_Fe and S_GA_Cu are more cathodic (0.117 V and 0.082 V versus SHE, respectively) The exchanged electron number is about 3.5–4.0 for all metal-doped materials. S_GA, with the lowest onset potential (0.044 V versus SHE) and an exchanged electron number of ~2.7 (suggesting a predominant formation of peroxides) is the worst catalyst. Similar results on iron-doped samples have been obtained in a previous work by Liang et al. [41] on Fe–N-decorated hybrids of CNTs grown on hierarchically porous carbon. However, their proposed synthetic path is more complicated and time-consuming than the one used here. As a general behavior, iron-doped carbons (S_GA_Fe and S_GA_FeCu) exhibit better electrocatalytic properties with respect to S_GA_Cu. This could be justified by the presence both of iron species [15] and of a large amount of N-CNTs characterized by a high electrocatalytic activity towards ORRs [42,43,44,45,46]. This peculiarity of N-CNTs derives from the presence of substitutional nitrogen in their graphene planes, which, disturbing their uniform π-cloud [47] and increasing the localized density of states at the Fermi level [48], imparts a n-type dopant activity. As a consequence, their specific electronic properties improve and their electrocatalytic activity increases [42,43,44,45,46,47,48,49]. However, the presence of both iron and N-CNTs cannot account by itself for the improved electrocatalytic activity of S_GA_FeCu with respect to S_GA_Fe.

As reported in [20], the surface hydrophilicity of active carbons plays an important role in their electroactivity toward ORRs. Therefore, the hydrophilicity of the present samples was measured in order to assess its influence on their performance. Since the contact angle measurements on rough materials are very critical [50], following [20], the time necessary to spread completely a water drop onto the sample surface was used to quantify the hydrophilicity (the shorter this time, the more hydrophilic the material). Hydrophilicity was found to increase in the order S_GA < S_GA_Fe < S_GA_Cu < S_GA_FeCu (Table 5).

Since Fe(0) is more hydrophilic than Cu(0) [49] and N-CNTs favorably interact with hydrophilic molecules [51], the experimental hydrophilicity scale (Table 5) suggests that the presence of a metal phase (as Cu(0)) is more influential on hydrophilicity than the presence of N-CNTs. Moreover, since all the metal-doped samples exhibit similar amounts of surface oxygen and nitrogen, forming typical hydrophilic groups on the surface, the hydrophilicity of the active site, rather than the entire surface, should be responsible for the different hydrophilicities of carbons.

Coming back to the different electrocatalytic activity of S_GA_Fe and S_GA_FeCu carbons, our physico-chemical characterization shows that they have very similar structure and properties but there is a larger hydrophilicity in the S_GA_FeCu sample, which is probably related to the presence of Cu(0) phase. Thus, we propose that the improved performance of S_GA_FeCu has to be attributed to the different hydrophilicity of its active centers. Actually, an increase of the interactions between water and active centers could favor interactions with molecular oxygen and thus, its adsorption and, finally, its reduction. Although the influence of the active center’s hydrophilicity on ORR activity of Pt-free materials should be deeply investigated, the results here presented pave the way to the comprehension of their properties.

## 4. Conclusions

In this work, catalysts based on nitrogen-modified carbons doped with different metals (Fe, Cu, and a mixture of them) were synthesized and characterized by BET, synchrotron radiation XRPD, micro-Raman, XPS, SEM, STEM, and hydrophilicity measurements. The N-modified carbon phases are characterized by porous structures and by the presence of N-CNTs. In the Fe-doped samples (S_GA_Fe and S_GA_FeCu), N-CNTs are more well-organized, in a larger amount, and longer than in the copper-doped ones. In Cu-doped (S_GA_Cu and S_GA_FeCu) samples, copper is present in the crystalline (ccp) phase.

In spite of the different nature of the metal-doping agent, catalysts exhibit few differences in terms of surface area, functional surface groups, and graphitization degree of the carbon phase. Electrocatalytic activity toward ORRs increases in the order S_GA_Cu < S_GA_Fe < S_GA_FeCu. The presence of a larger amount of N-CNTs can account for the better performance of iron-doped catalysts with respect to S_GA_Cu. Instead, also the hydrophilicity of the active sites, which decreases in the order S_GA_FeCu > S_GA_Cu > S_GA_Fe, must be considered in order to explain the higher electrocatalytic activity of S_GA_FeCu with respect to S_GA_Fe.

The presence of iron, N-CNTs, and an improved hydrophilicity synergically boost the electrocatalytic properties of ORR catalysts.

The preliminary results here presented highlight the influence of the hydrophilicity of active sites on ORRs and pave the way to the comprehension of the electrocatalytic properties of Pt-free materials.

## Figures and Tables

**Table 1:** Specific Surface Area (SSA) and Pore Area Distribution.

Sample Name	SSA/m^2^ g^−1^	Pores% *d* < 2 nm	Pores% 2 < *d* < 5 nm	Pores% 5 < *d* < 20 nm	Pores% *d* > 20 nm
S_GA	523	7.2	53.7	34.5	4.6
S_GA_Cu	556	3.9	56.3	35.6	4.2
S_GA_Fe	598	5.7	56.9	33.2	4.2
S_GA_FeCu	602	5.0	54.7	35.7	4.6

*d* = Pore diameter.

**Table 2:** Samples’ Surface Atomic Composition *.

Sample Name	%C	%N	%O	%Fe	%Cu
S_GA	88.6 ± 0.7	8.3 ± 0.5	3.1 ± 0.6	-	-
S_GA_Cu	88.0 ± 0.5	6.9 ± 0.9	4.7 ± 1.3	-	0.4 ± 0.2
S_GA_Fe	88.6 ± 0.5	7.1 ± 0.5	4.0 ± 0.5	0.3 ± 0.2	-
S_GA_FeCu	89.4 ± 1.2	7.1 ± 0.5	3.0 ± 1.0	0.3 ± 0.2	0.2 ± 0.2

* The values are averaged out of three replicates. Error is expressed as the larger value between the error associated with a single quantification (0.2% for Cu and Fe, 0.5% for other elements) and one standard deviation.

**Table 3:** Relative Peak Areas (RPA%) of N1s peaks *.

Peak Number	Binding Energies/eV	Functional Group	S_GA	S_GA_Cu	S_GA_Fe	S_GA_FeCu
(1)	398.3–398.5	Pyridinic N	25 ± 1	26 ± 2	29 ± 1	27 ± 2
(2)	399.2–399.6	N_x_-Me or amine	13 ± 4	16 ± 4	13 ± 1	14 ± 1
(3)	400.9–401.0	Pyrrolic N	37 ± 2	35 ± 2	36 ± 1	31 ± 4
(4)	402.0–403.0	Quaternary N	12 ± 3	10 ± 2	10 ± 1	11 ± 3
(5)	403.3–403.6	Graphitic N	6 ± 1	5 ± 1	5 ± 1	7 ± 1
(6)	404.8–405.1	Shake-up π-π *	3 ± 2	4 ± 2	5 ± 1	6 ± 1
(7)	406.8–406.9	Shake-up π-π *	4 ± 1	4 ± 1	2 ± 1	4 ± 1

* The error is expressed as the larger value between the error associated with a single curve-fitting procedure (1%, at worst) and one standard deviation averaged out of three replicates.

**Table 4:** ORR Onset *E*_onset_ (at *j* = 1 mA cm^−2^), Half Wave Potentials *E*_1/2_, and Exchanged Electrons Number n*e^−^*.

Sample Name	*E_onset_* versus SHE/V at *j* = 1 mA cm^−2^	*E*_1/2_ versus SHE/V	n *e^−^*
S_GA	0.044	0.033	2.72 ± 0.01
S_GA_Cu	0.082	0.068	3.82 ± 0.03
S_GA_Fe	0.117	0.066	3.98 ± 0.03
S_GA_FeCu	0.145	0.099	3.48 ± 0.02
Pt EC20	0.135	0.088	4.0 ± 0.1

**Table 5:** Time Necessary to Spread a Water Drop on a Surface.

Sample Name	Time/s
S_GA	7.9
S_GA_Fe	5.8
S_GA_Cu	4.7
S_GA_FeCu	4.0

## Supplementary Materials

The following are available online at http://www.mdpi.com/2079-4991/8/9/643/s1, Figure S1: Porosity Distribution. Figure S2: Results of Raman spectra decomposition. Figure S3: XPS survey of S_GA_FeCu. Figure S4: XPS N1s region of S_GA_FeCu. Figure S5: Superimposition of XPS C1s spectra. Figure S6: XPS Fe2p spectra of S_GA_FeCu and S_GA_Fe. Table S1: Parameters inferred from Raman spectra fitting. Table S2: Lattice parameters, weight fraction (WF), average displacement parameters (Umean), and fit residuals for the refinements performed on crystalline phases.

## References

[1] Greeley J., Stephens I.E.L., Bondarenko A.S., Johansson T.P., Hansen H.A., Jaramillo T.F., Rossmeisl J., Chorkendorff I., Nørskov J.K. (2009). Alloys of Platinum and Early Transition Metals as Oxygen Reduction Electrocatalysts. Nat. Chem..

[2] Wu G., Zelenay P. (2013). Nanostructured Nonprecious Metal Catalysts for Oxygen Reduction Reaction. Acc. Chem. Res..

[3] Ghosh T., Vukmirovic M.B., Di Salvo F.J., Adzic R.R. (2010). Intermetallics as Novel Supports for Pt Monolayer O_2_ Reduction Electrocatalysts: Potential for Significantly Improving Properties. J. Am. Chem. Soc..

[4] Bashyam R., Zelenay P. (2006). A Class of Non-Precious Metal Composite Catalysts for Fuel Cells. Nature.

[5] Lefèvre M., Proietti E., Jaouen F., Dodelet J.P. (2009). Iron-Based Catalysts with Improved Oxygen Reduction Activity in Polymer Electrolyte Fuel Cells. Science.

[6] Jaouen F., Herranz J., Lefèvre M., Dodelet J.-P., Kramm U.I., Herrmann I., Bogdanoff P., Maruyama J., Nagaoka T., Garsuch A. (2009). Cross-Laboratory Experimental Study of Non-Noble-Metal Electrocatalysts for the Oxygen Reduction Reaction. ACS Appl. Mater. Interfaces.

[7] Wu G., More K.L., Johnston C.M., Zelenay P. (2011). High-Performance Electrocatalysts for Oxygen Reduction Derived from Polyaniline, Iron, and Cobalt. Science.

[8] Cheon J.Y., Kim T., Choi Y., Jeong H.Y., Kim M.G., Sa Y.J., Kim J., Lee Z., Yang T.H., Kwon K. (2013). Ordered Mesoporous Porphyrinic Carbons with Very High Electrocatalytic Activity for the Oxygen Reduction Reaction. Sci. Rep..

[9] Guan B.Y., Yu L., Lou X.W. (2016). A Dual-Metal–Organic-Framework Derived Electrocatalyst for Oxygen Reduction. Energy Environ. Sci..

[10] Xia B.Y., Yan Y., Li N., Wu H.B., Lou X.W., Wang X. (2016). A Metal–Organic Framework-Derived Bifunctional Oxygen Electrocatalyst. Nat. Energy.

[11] Liu T., Zhao P., Hua X., Luo W., Chen S., Cheng G. (2016). An Fe–N–C Hybrid Electrocatalyst Derived from a Bimetal–Organic Framework for Efficient Oxygen Reduction. J. Mater. Chem. A.

[12] Masa J., Xia W., Muhler M., Schuhmann W. (2015). On the Role of Metals in Nitrogen-Doped Carbon Electrocatalysts for Oxygen Reduction. Angew. Chem. Int. Ed..

[13] Tylus U., Jia Q., Strickland K., Ramaswamy N., Serov A., Atanassov P., Mukerjee S. (2014). Elucidating Oxygen Reduction Active Sites in Pyrolyzed Metal-Nitrogen Coordinated Non-Precious-Metal Electrocatalyst Systems. J. Phys. Chem. C.

[14] Choi C.H., Choi W.S., Kasian O., Mechler A.K., Sougrati M.T., Brüller S., Strickland K., Jia Q., Mukerjee S., Mayrhofer K.J.J. (2017). Unraveling the Nature of Sites Active toward Hydrogen Peroxide Reduction in Fe-N-C Catalysts. Angew. Chem. Int. Ed..

[15] Workman M.J., Dzara M., Ngo C., Pylypenko S., Serov A., McKinney S., Gordon J., Atanassov P., Artyushkova K. (2017). Platinum Group Metal-Free Electrocatalysts: Effects of Synthesis on Structure and Performance in Proton-Exchange Membrane Fuel Cell Cathodes. J. Power Sources.

[16] Longhi M., Marzorati S., Checchia S., Sacchi B., Santo N., Zaffino C., Scavini M. (2016). Sugar-Based Catalysts for Oxygen Reduction Reaction. Effects of the Functionalization of the Nitrogen Precursors on the Electrocatalytic Activity. Electrochim. Acta.

[17] Su C.-Y., Cheng H., Li W., Liu Z.-Q., Li N., Hou Z., Bai F.-Q., Zhang H.-X., Ma T.-Y. (2017). Atomic Modulation of FeCo–Nitrogen–Carbon Bifunctional Oxygen Electrodes for Rechargeable and Flexible All-Solid-State Zinc–Air Battery. Adv. Energy Mater..

[18] Witkowska A., Giuli G., Renzi M., Marzorati S., Yiming W., Nobili F., Longhi M. (2015). Fe Local Structure in Pt-Free Nitrogen-Modified Carbon Based Electrocatalysts: XAFS Study. J. Phys. Conf. Ser..

[19] Artyushkova K., Serov A., Rojas-Carbonell S., Atanassov P. (2015). Chemistry of Multidinous Active Sites for Oxygen Reduction Reaction in Transition Metal-Nitrogen-Carbon Electrocatalysts. J. Phys. Chem. C.

[20] Hao G.-P., Sahraie N.R., Zhang Q., Krause S., Oschatz M., Bachmatiuk A., Strasser P., Kaskel S. (2015). Hydrophilic Non-Precious Metal Nitrogen-Doped Carbon Electrocatalysts for Enhanced Efficiency in Oxygen Reduction Reaction. Chem. Commun..

[21] Kumar K.V., Preuss K., Guo Z.X., Titirici M.M. (2016). Understanding the Hydrophilicity and Water Adsorption Behavior of Nanoporous Nitrogen-Doped Carbons. J. Phys. Chem. C.

[22] Bogdanov K., Fedorov A., Osipov V., Enoki T., Takai K., Hayashi T., Ermakov V., Moshkalev S., Baranov A. (2014). Annealing-Induced Structural Changes of Carbon Onions: High-Resolution Transmission Electron, Microscopy and Raman Studies. Carbon.

[23] Larson A.C., Von Dreele R.B. (2004). General Structural Analysis System (GSAS).

[24] Sing K.S.W., Everett D.H., Haul R.A.W., Moscou L., Pierotti R.A., Rouquerol J., Siemieniewska T. (1985). Reporting Physisorption Data for Gas/Solid Systems with Special Reference to the Determination of Surface Area and Porosity. Pure Appl. Chem..

[25] Thommes M. (2010). Physical Adsorption Characterization of Nanoporous Materials. Chem. Ing. Tech..

[26] Leonard N.D., Nallathambi V., Calabrese Burton S. (2011). Carbon Supports for Non-Precious Metal Proton Exchange Membrane Fuel Cells. ECS Trans..

[27] Xy J.B., Zhao T.S. (2013). Mesoporous Carbon with Uniquely Combined Electrochemical and Mass Transport Characteristics for Polymer Electrolyte Membrane Fuel Cells. RSC Adv..

[28] Marzorati S., Longhi M. (2016). Templating Induced Behavior of Platinum-free Carbons for Oxygen Reduction Reaction. J. Electroanal. Chem..

[29] Ferrari A.C., Robertson J. (2001). Interpretation of Raman Spectra of Disordered and Amorphous Carbon. Phys. Rev. B.

[30] Santangelo S. (2016). Controlled Surface Functionalisation of Carbon Nanotubes by Nitric Acid Vapors Generated from Sub-Azeotropic Solution. Surf. Interface Anal..

[31] Cançado L.G., Takai K., Enoki T., Endo M., Kim Y.A., Mizusaki H., Jorio A., Coelho L.N., Magalhães-Paniago R., Pimenta M.A. (2006). General Equation for the Determination of the Crystallite Size L_a_ of Nanographite by Raman Spectroscopy. Appl. Phys. Lett..

[32] Klement W. (1963). Technical Report 401844.

[33] Chen Q., Jin Z. (1995). The Fe-Cu System: A Thermodynamic Evaluation. Metal. Mater. Trans. A.

[34] Peng H., Liu F., Liu X., Liao S., You C., Tian X., Nan H., Luo F., Song H., Fu Z. (2014). Effect of Transition Metals on the Structure and Performance of the Doped Carbon Catalysts Derived from Polyaniline and Melamine for ORR Application. ACS Catal..

[35] Lahaye J., Nanse G., Bagreev A., Strelko V. (1999). Porous Structure and Surface Chemistry of Nitrogen Containing Carbons from Polymers. Carbon.

[36] Díaz J., Paolicelli G., Ferrer S., Comin F. (1996). Separation of the sp^3^ and sp^2^ Components in the C1s Photoemission Spectra of Amorphous Carbon Films. Phys. Rev. B.

[37] Biesinger M.C., Lau L.W.M., Gerson A.R., Smart R.S.C. (2010). Resolving Surface Chemical States in XPS Analysis of First Row Transition Metals, Oxides and Hydroxides: Sc, Ti, V, Cu, and Zn. Appl. Surf. Sci..

[38] Galbiati I., Bianchi C.L., Longhi M., Carra A., Formaro L. (2010). Iron and Copper Containing Oxygen Reduction Catalysts from Templated Glucose-Histidine. Fuel Cells.

[39] Formaro L., Longhi M., Messina P., Galbiati I. (2014). Catalysts Free from Noble Metals Suitable for the Electrochemical Reduction of Oxygen. U.S. Patent.

[40] Andrews R., Jacques D., Qian D., Rantell T. (2002). Multiwall Carbon Nanotubes: Synthesis and Application. Acc. Chem. Res..

[41] Liang J., Zhou R.F., Chen X.M., Tang Y.H., Qiao S.Z. (2014). Fe-N Decorated Hybrids of CNTs Grown on Hierarchically Porous Carbon for High-Performance Oxygen Reduction. Adv. Mater..

[42] Maldonado S., Stevenson K.J. (2005). Influence of Nitrogen Doping on Oxygen Reduction Electrocatalysis at Carbon Nanofiber Electrodes. J. Phys. Chem. B.

[43] Van Dommele S., de Jong K.P., Bitter J.H. (2006). Nitrogen-Containing Carbon Nanotubes as Solid Base Catalysts. Chem. Commun..

[44] Shao Y., Sui J., Yin G., Gao Y. (2008). Nitrogen-Doped Carbon Nanostructures and their Composites as Catalytic Materials for Proton Exchange Membrane Fuel Cell. Appl. Catal. B.

[45] Sidik R.A., Anderson A.B., Subramanian N.P., Kumaraguru S.P., Popov B.N. (2006). O_2_ Reduction on Graphite and Nitrogen-Doped Graphite: Experiment and Theory. J. Phys. Chem. B.

[46] Liu R., Wu D., Feng X., Müllen K. (2010). Nitrogen-Doped Ordered Mesoporous Graphitic Arrays with High Electrocatalytic Activity for Oxygen Reduction. Angew. Chem..

[47] Chizari K., Janowska I., Houllé M., Florea I., Ersen O., Romero T., Bernhardt P., Ledoux M.J., Pham-Huu C. (2010). Tuning of Nitrogen-Doped Carbon Nanotubes as Catalyst Support for Liquid-Phase Reaction. Appl. Catal. A Gen..

[48] Terrones M., Ajayan P.M., Banhart F., Blase X., Carroll D.L., Charlier J.C., Czerw R., Foley B., Grobert N., Kamalakaran R. (2002). N-Doping and Coalescence of Carbon Nanotubes: Synthesis and Electronic Properties. Appl. Phys. A Mater. Sci. Process..

[49] Ismagilov Z.R., Shalagina A.E., Podyacheva O.Y., Ischenko A.V., Kibis L.S., Boronin A.I., Chesalov Y.A., Kochubey D.I., Romanenko A.I., Anikeeva O.B. (2009). Structure and Electrical Conductivity of Nitrogen-Doped Carbon Nanofibers. Carbon.

[50] Afanasev B.N., Akulova Y.P. (2000). A Correlation Between the Hydrophilicity of a Metal and its Surface Tension. Calculation of the Bond Energy of Water Molecules Adsorbed on an Uncharged Metal Surface. Prot. Met..

[51] Lee W.J., Maiti U.N., Lee J.M., Lim J., Han T.H., Sang O.K. (2014). Nitrogen-Doped Carbon Nanotubes and Graphene Composite Structures for Energy and Catalytic Applications. Chem. Commun..

