# Supplementary material for: Synergistic Effects of Active Sites’ Nature and Hydrophilicity on the Oxygen Reduction Reaction Activity of Pt-Free Catalysts
Source: Nanomaterials (Basel). 2018 Aug 22;8(9):643. doi: 10.3390/nano8090643 (PMC6163935; doi:10.3390/nano8090643)
Supplement: Supplementary file 1 [file nanomaterials-08-00643-s001.pdf]

Supplementary material

Synergistic Effects of Active Sites Nature and Hydrophilicity on Oxygen Reduction Reaction Activity of Pt-Free Catalysts.

Mariangela Longhi,[a,*] Camilla Cova,[a] Eleonora Pargoletti,[a] Mauro Coduri,[b] Saveria Santangelo,[c] Salvatore Patanè,[d] Nicoletta Ditaranto,[e] Nicola Cioffi,[e] Anna Facibeni,[f] Marco Scavini[a]

[a]Università degli Studi di Milano, Dipartimento di Chimica, Via Golgi 19, 20133 Milano (Italy)

[b]ESRF - The European Synchrotron, 71, Avenue des Martyrs, 38043 Grenoble (France)

[c]Università “Mediterranea”, Dipartimento di Ingegneria Civile, dell’Energia, dell’Ambiente e dei Materiali (DICEAM), Via Graziella, Loc. Feo di Vito, 89122 Reggio Calabria (Italy)

[d]Università di Messina, Dipartimento di Scienze Matematiche e Informatiche, Scienze Fisiche e Scienze della Terra (MIFT), Viale Stagno d’Alcontres 31, 98166 Messina (Italy)

[e]Università degli Studi di Bari "Aldo Moro", Dipartimento di Chimica, Via Orabona 4, 70125 Bari (Italy)

[f]Politecnico di Milano, Dipartimento di Energia and NEMAS - Centre for NanoEngineered MAterials and Surfaces, Via Ponzio 34/3, 20133, Milano (Italy)

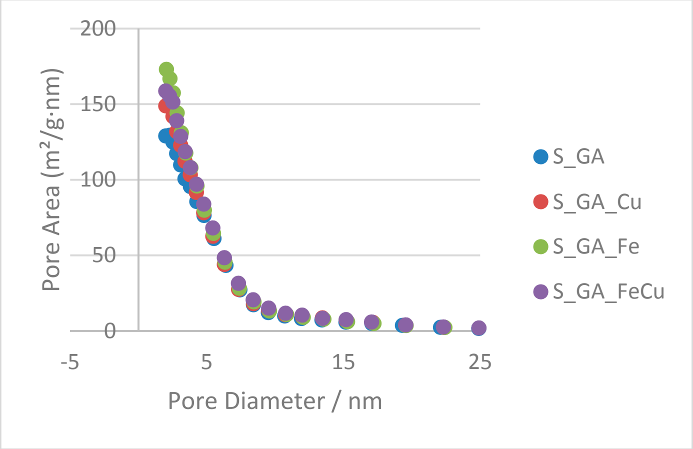

Figure S1. Porosity Distribution

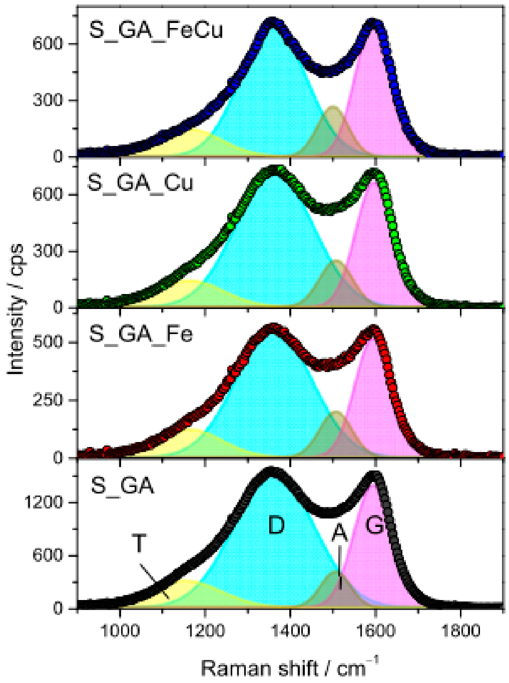

Figure S2. Results of Raman spectra decomposition. The low-frequency region of the spectra (< 2000 cm^{-1}) is fitted to four bands [S1], namely the T-band (~1160 cm^{-1}), due to trans-poly-acetylene-like chains formed at the zigzag edges of the defective graphitic layers, the D-band (~1360 cm^{-1}), generated by finite size effects and by lattice defects breaking the translational symmetry of graphitic layers, the A-band (~1500 cm^{-1}), associated to amorphous phases connected to the ordered graphene planes through C*sp*[3] bonds, and the G-band (~1590 cm^{-1}), originating from the stretching of C=C pairs.

Table S1: Parameters inferred from Raman spectra fitting. (a) Center frequency positions (ω) and widths (γ, namely FWHM) of the main bands. (b) Relative to G-band intensity of the X-band (I_X/I_G), calculated as integrated intensity ratio. The graphitization index [S2], I_G/I_D, and the average size of the graphitic crystallites, estimated from I_G/I_D as $L_C = 560\cdot(I_G/I_D)\cdot E_L^{-4}$ (with E_L=2.33 eV denoting the excitation laser energy) [S3], are also reported.

(a)

Sample	ω_T / cm^{-1}	γ_T / cm^{-1}	ω_D / cm^{-1}	γ_D / cm^{-1}	ω_A / cm^{-1}	γ_A / cm^{-1}	ω_G / cm^{-1}	γ_G / cm^{-1}
S_GA	1151	203	1363	224	1508	83	1597	109
S_GA_Cu	1165	187	1364	215	1510	92	1599	103
S_GA_Fe	1164	180	1363	211	1509	90	1597	102
S_GA_FeCu	1163	181	1362	183	1502	86	1596	101

(b)

Sample	I_T/I_G	A_D/A_G	I_A/I_G	I_G/I_D	L_C / nm
S_GA	0.41	2.27	0.22	0.44	8.4
S_GA_Cu	0.38	2.26	0.34	0.44	8.4
S_GA_Fe	0.42	2.25	0.34	0.44	8.4
S_GA_FeCu	0.38	1.82	0.33	0.55	10.5

Table S2. Lattice parameters, weight fraction (WF), average displacement parameters (U_{mean}) and fit residuals for the refinements performed on crystalline phases.

	Sample	ZGAG_Cu	ZGAG_CuFe
Cu	Space group	Fm-3m	Fm-3m
	a/Å	3.61711(1)	3.61774(1)
	WF/%	2.7(1)	1.6(1)
C	Space group	P63mc	P63mc
	a/Å	2.481(2)	2.476(2)
	c/Å	6.928(5)	6.934(5)
	WF	97.3(1)	98.4(1)
	Umean/ Å2	0.0064(2)	0.0056(3)
	R(F2)	0.0368	0.0414
	RP	0.0270	0.0270

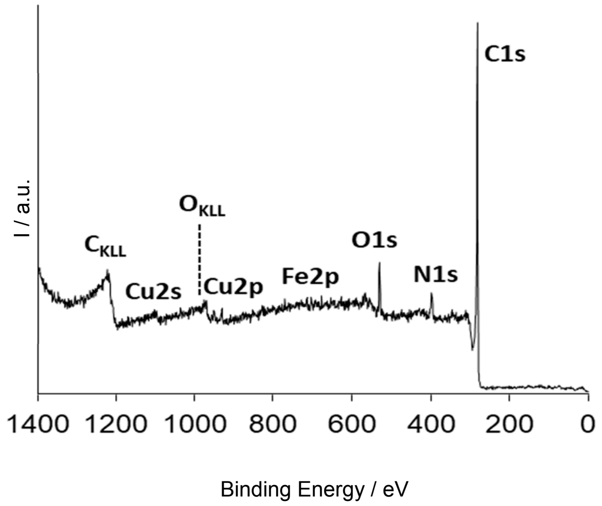

Figure S3. XPS survey of S_GA_FeCu.

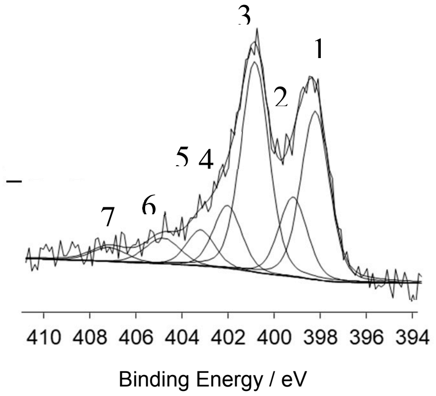

Figure S4. XPS N1s region of S_GA_FeCu. 1) Pyridinic N; 2) N_x-Me or Amine N; 3) Pyrrolic N; 4) Quaternary N; 5) Graphitic N; 6) Shake up π-π*; 7) Shake up π-π*

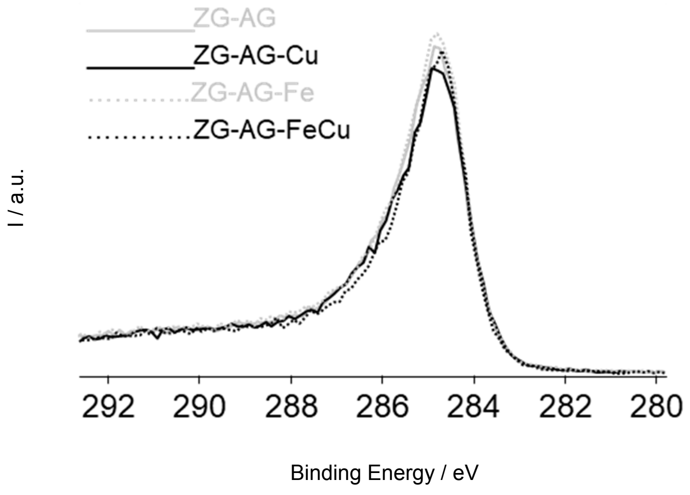

Figure S5. Superimposition of XPS C*1s* spectra

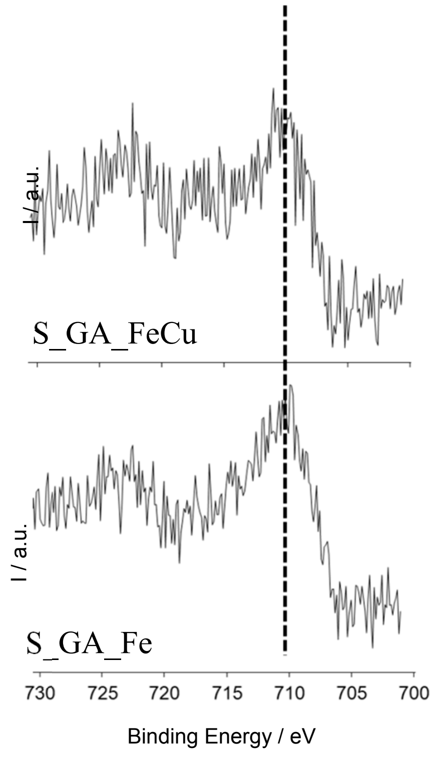

Figure S6. XPS Fe2*p* spectra of S_GA_FeCu and S_GA_Fe

References

[S1] K. Bogdanov, A. Fedorov, V. Osipov, T. Enoki, K. Takai, T. Hayashi, V. Ermakov, S. Moshkalev, A. Baranov, Annealing-Induced Structural Changes of Carbon Onions: High-Resolution Transmission Electron, Microscopy and Raman Studies, Carbon 73 (2014) 78–86. DOI: 10.1016/j.carbon.2014.02.041.

[S2] S. Santangelo, Functionalisation of Carbon Nanotubes by Nitric Acid Vapors Generated from Sub-Azeotropic Solution, Surf. Interf. Analysis 48 (2016) 17–25. DOI: DOI:10.1002/sia.5875.

[S3] L.G. Cançado, K. Takai, T. Enoki, M. Endo, Y.A. Kim, H. Mizusaki, A. Jorio, L.N. Coelho, R. Magalhães-Paniago, M.A. Pimenta, General Equation for the Determination of the Crystallite Size L_a of Nanographite by Raman Spectroscopy, Appl. Phys. Lett. 88 (2006) 163106−163108. DOI: 10.1063/1.2196057.
